# ﻿Three new species of the spider genus *Liphistius* (Araneae, Mesothelae, Liphistiidae) from Thailand

**DOI:** 10.3897/zookeys.1104.83264

**Published:** 2022-06-10

**Authors:** Yi Zhan, Varat Sivayyapram, Fengxiang Liu, Daiqin Li, Xin Xu

**Affiliations:** 1 College of Life Sciences, Hunan Normal University, 36 Lushan Road, Changsha 410081, Hunan Province, China; 2 Center of Excellence in Entomology and Department of Biology, Faculty of Science, Chulalongkorn University, Bangkok 10330, Thailand; 3 State Key Laboratory of Biocatalysis and Enzyme Engineering, School of Life Sciences, Hubei University, 368 Youyi Road, Wuhan 430062, Hubei Province, China; 4 Centre for Behavioural Ecology and Evolution (CBEE), School of Life Sciences, Hubei University, 368 Youyi Road, Wuhan 430062, Hubei Province, China; 5 Department of Biological Sciences, National University of Singapore, 14 Science Drive 4, 117543, Singapore, Singapore

**Keywords:** Morphology, Southeast Asia, taxonomy, trapdoor spiders

## Abstract

We diagnose and describe three new species of the primitively segmented spider genus *Liphistius* from Thailand, based on male palp and female genital morphology: *L.hatyai* Zhan & Xu, **sp. nov.** (♂♀), *L.keeratikiati* Zhan & Xu, **sp. nov.** (♂♀), and *L.inthanon* Zhan & Xu, **sp. nov.** (♂♀). The classification of the three new species of *Liphistius* is discussed: *L.hatyai***sp. nov.** and *L.keeratikiati***sp. nov.** are assigned to the *trang*-group, and *L.inthanon***sp. nov.** is placed in the *bristowei*-group according to male palp and female genital morphology.

## ﻿Introduction

**Figure 1.**
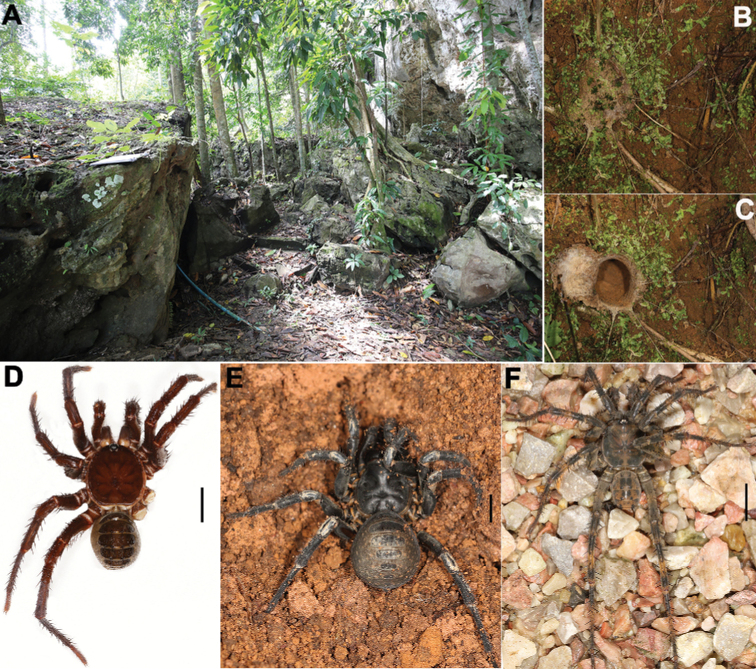
Microhabitat, burrows, and general somatic morphology of three new *Liphistius* species **A** microhabitat **B** burrow with trapdoor closed **C** same, trapdoor opened **D** male, *L.hatyai* Zhan & Xu, sp. nov (XUX-2017-492) **E** female, *L.inthanon* Zhan & Xu, sp. nov. (XUX-2017-374) **F** male, *L.keeratikiati* Zhan & Xu, sp. nov. (XUX-2017-435) Scale bars: 5 mm (**D, E, F**).

As the sister lineage to all other extant spiders, the primitively segmented spider family Liphistiidae, belonging to the suborder Mesothelae, retains some plesiomorphic characters, such as abdominal tergites (Fig. 1) and spinnerets situated on the median area of the ventral abdomen (Platnick and Gertsch 1976; Coddington and Levi 1991; Haupt 2003). Currently, Liphistiidae contains 166 species belonging to eight genera in two subfamilies, Liphistiinae Thorell, 1869 and Heptathelinae Kishida, 1923 (WSC 2022). The subfamily Liphistiinae containing a single genus, *Liphistius* Schiødte, 1849, occurs in China (Yunnan Province), Indonesia (Sumatra), Laos, Peninsular Malaysia, Myanmar, and Thailand (WSC 2022).

The genus *Liphistius* was erected by Schiødte (1849) based on the type species *Liphistiusdesultor* found in Malaysia (Schiødte 1849). Since then, an increasing number of *Liphistius* species have been described from Asia. Currently, *Liphistius* includes 59 known species, of which 33 are known from Thailand (WSC 2022). Platnick and Sedgwick (1984) presented the first taxonomic revision of the genus by describing 14 species from Indonesia (Sumatra), Peninsular Malaysia, Myanmar, and Thailand. Recently, Schwendinger and colleagues provided taxonomic revisions of *Liphistius* in Peninsular Malaysia (Schwendinger 2017; Schwendinger et al. 2019).

Members of *Liphistius* can be divided into seven species-groups based on male and female genital morphology: the *batuensis*-group, *birmanicus*-group, *bristowei*-group, *linang*-group, *malayanus*-group, *trang*-group, and *tioman*-group (Schwendinger 1990, 2017; Schwendinger et al. 2019). Specifically, the *trang*-group is subdivided into six species complexes (Schwendinger 1990, 1996, 1998; Schwendinger et al. 2019). Out of 33 named *Liphistius* species from Thailand, 32 are assigned to four species-groups, and one (*L.jarujini* Ono, 1988) is an *incertae sedis* species: *trang*-group (25 species), *bristowei*-group (5 species), *birmanicus*-group (1 species), *linang*-group (1 species) (for details see Sivayyapram et al. 2017).

To investigate the species diversity of *Liphistius* in Thailand, we carried out several field trips in the country. After examining specimens collected, here we diagnose and describe three new *Liphistius* species based on the genital morphology of both sexes.

## ﻿Material and methods

**Figure 2.**
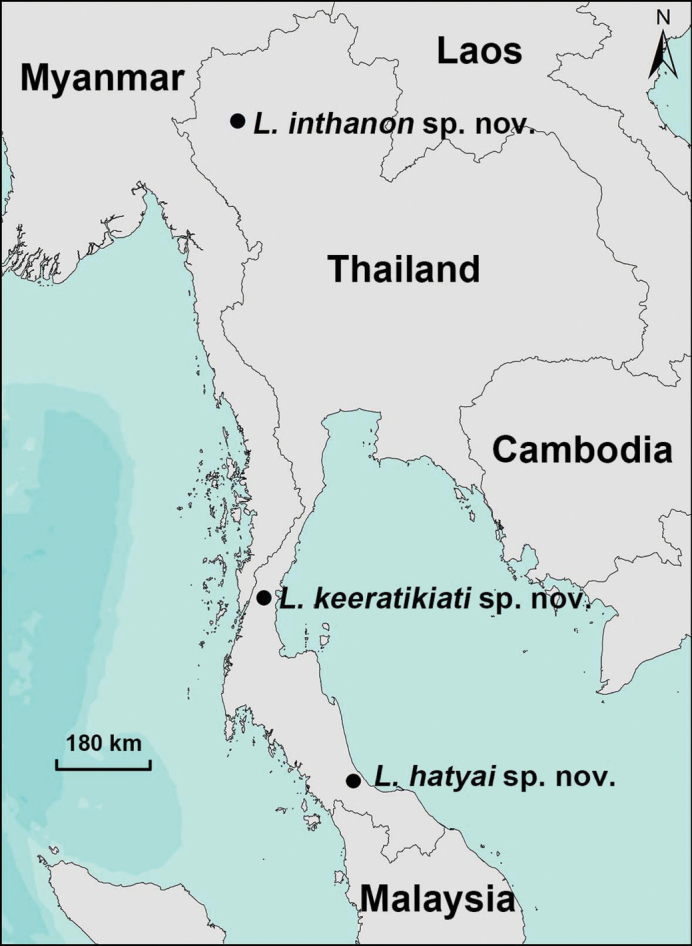
Map of Thailand showing the localities of three new *Liphistius* species described.

All specimens were collected in Thailand (Fig. 2). We removed the right four legs of adults, preserved in 100% ethanol and kept at –80 °C for extracting genome DNA. We preserved specimen in 80% ethanol as the voucher for morphological examination. All the voucher specimens are deposited at the College of Life Sciences, Hunan Normal University, Changsha, Hunan Province, China.

We examined and dissected the specimens using an Olympus SZ51 stereomicroscope. The soft tissues of female genitalia were degraded using 10 mg/ml pancreatin for at least 3 h at room temperature. We used a digital camera CCD mounted on an Olympus BX53 compound microscope to photograph male palp and female genitalia, and then generated compound focused images using Helicon Focus v. 6.7.1. All measurements were carried out under a Leica M205C stereomicroscope using the software of Leica Application Suite v. 4 and are given in millimeters. Palp and leg measurements are given in the following order: leg total length (femur + patella + tibia + metatarsus [absent on palp] + tarsus).

### ﻿Abbreviations used in the text

**ALE** anterior lateral eyes;

**AME** anterior median eyes;

**PLE** posterior lateral eyes;

**PME** posterior median eyes;

**BL** body length (excluding chelicerae);

**CL** carapace length;

**OL** opisthosoma length;

**CW** carapace width;

**OW** opisthosoma width.

## ﻿Taxonomy

**Family Liphistiidae Thorell, 1869**

### ﻿Subfamily Liphistiinae Thorell, 1869

#### ﻿Genus Liphistius

Taxon classificationAnimaliaAraneaeLiphistiidae

Schiødte, 1849

30B6911F-0F85-5289-8B73-4FA8E015003C

##### Type species.

*Liphistiusdesultor* Schiødte, 1849.

##### Diagnosis.

**Figure 3.**
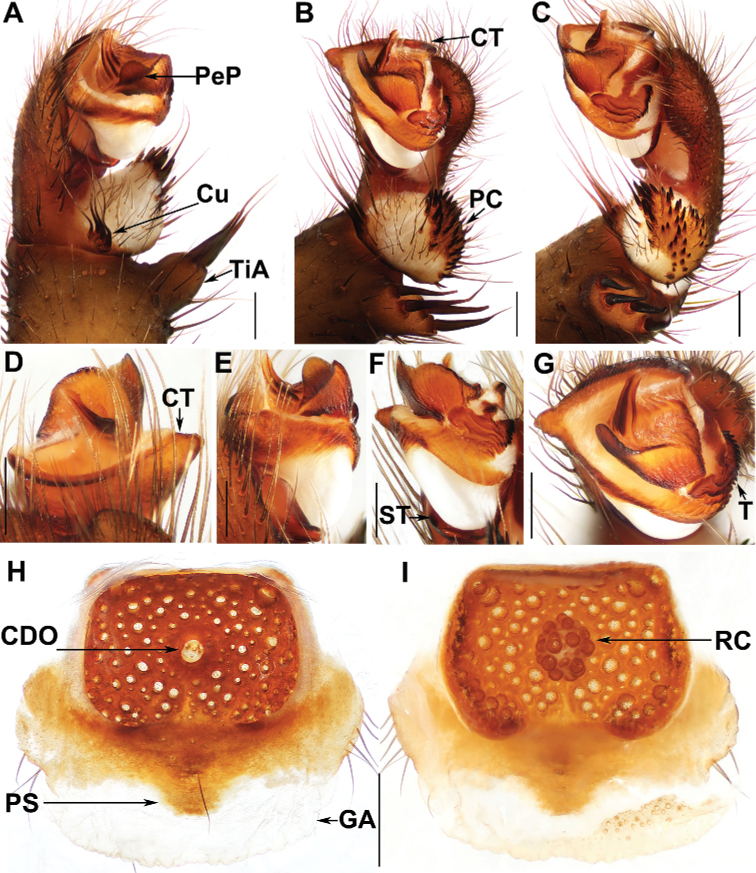
Male palp and female genitalia of *Liphistiushatyai* Zhan & Xu, sp. nov. **A** palp, prolateral view **B** palp, ventral view **C** palp, retrolateral view **D–G** palp, distal views **H** vulva, dorsal view **I** vulva, ventral view **A–G** XUX-2017-492 (holotype) **H, I** XUX-2017-493. Abbreviations used: CDO = central dorsal opening; GA = genital atrium; PS = posterior stalk; RC = receptacular cluster; CT = contrategulum; Cu = cumulus; PC = paracymbium; PeP = paraembolic plate; ST = subtegulum; T = tegulum; TiA = tibial apophysis. Scale bars: 0.5 mm.

**Figure 4.**
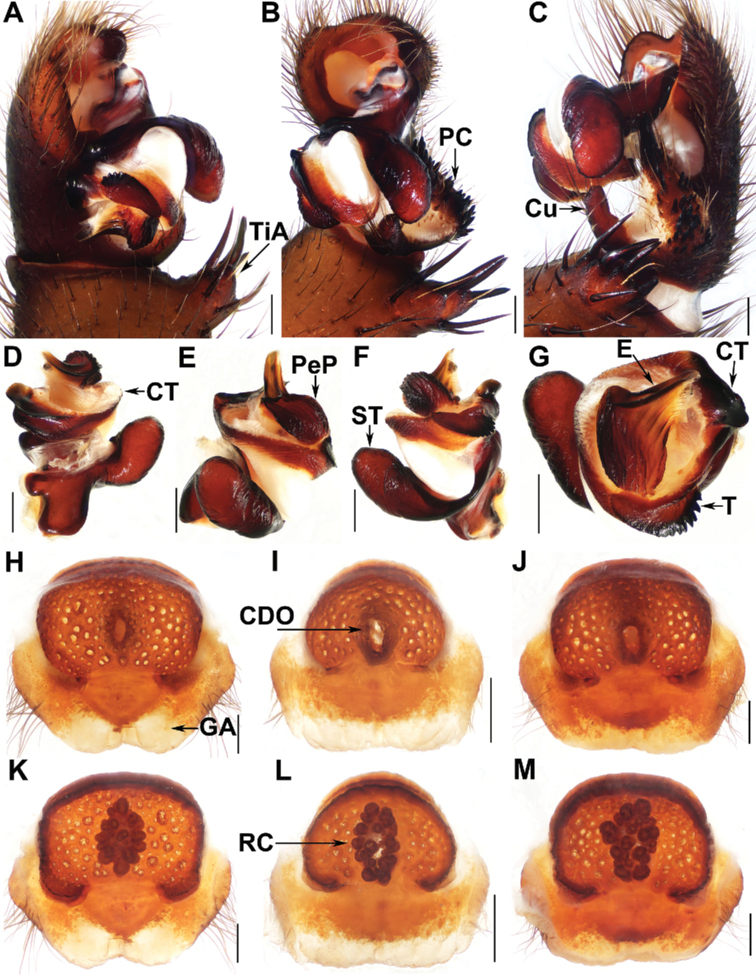
Male palp and female genitalia of *Liphistiusinthanon* Zhan & Xu, sp. nov. **A** palp, prolateral view **B** palp, ventral view **C** palp, retrolateral view **D–G** palp, distal views **H–J** vulva, dorsal view **K–M** vulva, ventral view **A–C** XUX-2017-372A (holotype) **D–G** XUX-2017-373A **H, K** XUX-2017-372 **I, L** XUX-2017-379 **J, M** XUX-2017-381. Abbreviations used: CDO = central dorsal opening; GA = genital atrium; RC = receptacular cluster; CT = contrategulum; Cu = cumulus; E = embolus; PC = paracymbium; PeP = paraembolic plate; ST = subtegulum; T = tegulum; TiA = tibial apophysis. Scale bars: 0.5 mm.

**Figure 5.**
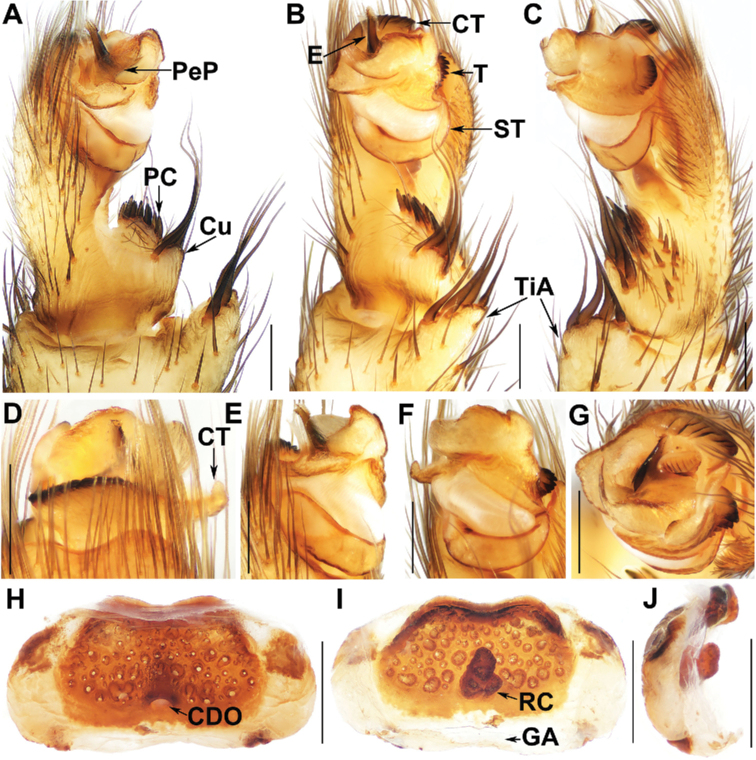
Male palp and female genitalia of *Liphistiuskeeratikiati* Zhan & Xu, sp. nov. **A** palp, prolateral view **B** palp, ventral view **C** palp, retrolateral view **D–G** palp, distal views **H** vulva, dorsal view **I** vulva, ventral view **J** vulva, lateral view **A–G** XUX-2017-439 (holotype) **H–J** XUX-2017-431. Abbreviations used: CDO = central dorsal opening; GA = genital atrium; RC = receptacular cluster; CT = contrategulum; Cu = cumulus; E = embolus; PC = paracymbium; PeP = paraembolic plate; ST = subtegulum; T = tegulum; TiA = tibial apophysis. Scale bars: 0.5 mm.

*Liphistius* differs from the other seven liphistiid genera by the presence of signal lines radiating from the burrow’s entrance (Fig. 1B, C), by the male palp having a tibial apophysis (Figs 3A, 4A, 5A), and by the female genitalia having a sclerotized poreplate and a median receptacular cluster (Figs 3H–I, 4H–M, 5H–J).

##### Distribution.

China (Yunnan Province), Indonesia (Sumatra), Laos, Peninsular Malaysia, Myanmar, and Thailand.

#### ﻿ Liphistius hatyai

Taxon classificationAnimaliaAraneaeLiphistiidae

Zhan & Xu sp. nov.

F35DBC37-BC2B-59A6-97C1-975D50E9F452

http://zoobank.org/0C8153A0-51E6-42A0-A009-BEDA4DB4D77E

Fig. 3

##### Type material.

***Holotype***: Thailand • 1 ♂; Songkhla Province, Hat Yai District, Kho Hong, 7.04°N, 100.50°E; alt. 25 m; 13 November 2016; N. Warrit, V. Sivayyapram, N. Chatthanabun, P. Traiyasut leg.; XUX-2017-492. ***Paratype***: Thailand • 1 ♀, same data as for the holotype; XUX-2017-493.

##### Diagnosis.

The male of *L.hatyai* sp. nov. resembles males of *L.albipes* Schwendinger, 1995 and *L.yangae* Platnick & Sedgwick, 1984 in having a rounded, scale-like paraembolic plate (Fig. 3A, E) but can be distinguished in having the tegulum with three transverse ridges in retrolateral view, while the latter two species have only one transverse ridge (Fig. 3C, G); from males of *L.bicoloripes* Ono, 1988, *L.castaneus* Schwendinger, 1995, and *L.niphanae* Ono, 1988 in having the cumulus slightly elevated (Fig. 3A–C); from the male of *L.inthanon* sp. nov. in having the tibial apophysis with fewer and longer setae (Fig. 3A–C), the cumulus slightly elevated (Fig. 3A–C), the subtegular apophysis absent (Fig. 3A, F), and the embolic parts detached (Fig. 3A–G); from the male of *L.keeratikiati* sp. nov. in having the sclerotised embolic part with three longitudinal ridges reaching the apex (Fig. 3A) and the tegulum with three transverse ridges distally (Fig. 3C, G); from males of other *Liphistius* species in having the cumulus slightly elevated, the sclerotised embolic part with three longitudinal ridges in prolateral view, and the subtegular apophysis absent (Fig. 3A, B, E).

The female of *L.hatyai* sp. nov. differs from females of *L.albipes* and *L.castaneus* in having a slightly narrower V-shaped posterior stalk (Fig. 3H, I); from females of *L.bicoloripes* and *L.castaneus* in having poreplate lacking anterolateral lobes (Fig. 3I); from the female of *L.niphanae* in having the anterior margin of the poreplate straight (Fig. 3H, I); from females of *L.yangae*, *L.inthanon* sp. nov., and *L.keeratikiati* sp. nov. in having the poreplate almost squared and with a slightly V-shaped posterior stalk (Fig. 3H, I); from females of other *Liphistius* species in having a hair at the center of posterior stalk dorsally (Fig. 3H).

##### Description.

**Male.** Carapace reddish-brown, with a few short, scattered bristles; opisthosoma brown, with 12 brown tergites, close to each other, 2–6 larger than others, fifth largest; chelicerae robust, promargin of cheliceral groove with 12 denticles of variable size; labium yellow and fused with sternum; sternum yellow, with a few short setae on the anterior tip and many long setae on the elongated posterior tip; legs yellowish brown, with strong hairs and spines, without distinct annulations, with 3 tarsal claws; 8 spinnerets. Measurements: BL 17.6, CL 8.81, CW 8.42, OL 8.33, OW 7.31; eye sizes and interdistances: AME 0.06, ALE 0.77, PME 0.47, PLE 0.59, AME–AME 0.13, AME–ALE 0.21, PME–PME 0.08, PME–PLE 0.09, ALE–PLE 0.12, ALE–ALE 0.17, PLE–PLE 0.39, AME–PME 0.12. Labium 0.55 long and 0.70 wide. Sternum 3.74 long and 1.04 wide. Leg I 22.55 (7.11 + 2.57 + 4.90 + 5.64 + 2.33), leg II 22.85 (6.76 + 1.73 + 5.56 + 6.06 + 2.74), leg III 26.62 (6.89 + 3.55 + 5.63 + 7.51 + 3.04), leg IV 30.54 (9.07 + 3.83 + 7.50 + 10.25 + 2.63).

***Palp***: tibial apophysis with four setae of same length, stouter basally and slender distally (Fig. 3A–C); paracymbium with many setae situated at tip (Fig. 3A–C); several tapering spines on slightly elevated cumulus (Fig. 3A–C); contrategulum with a triangular process, and an arched smooth edge with a sharp projection (Fig. 3B, C, G); tegulum with a serrated edge proximally and 3 transverse ridges distally (Fig. 3B, C, G); embolic parts detached (Fig. 3A–C, G), paraembolic plate semicircular, scale-like (Fig. 3A, E); embolus with 3 distinct longitudinal ridges reaching the tip prolaterally, with a few denticulations on apex, and with a flat membranous opening (Fig. 3A–C, G).

**Female.** Carapace orange, with few short, scattered bristles; opisthosoma gray, with 12 brown tergites, close to each other, with gray patches, 2–6 larger than others, fifth largest; 8 eyes on dark ocular tubercle; chelicerae robust, reddish brown; promargin of cheliceral groove with 10 denticles of variable size; labium yellow, fused with sternum; sternum yellow with several setae; legs with strong setae and spines, without distinct annulations, with 3 tarsal claws; 8 spinnerets. Measurements: BL 23.8, CL 11.42, CW 10.33, OL 11.03, OW 10.06; eye sizes and interdistances: AME 0.14, ALE 1.00, PME 0.52, PLE 0.61, AME–AME 0.12, AME–ALE 0.25, PME–PME 0.10, PME–PLE 0.12, ALE–PLE 0.15, ALE–ALE 0.10, PLE–PLE 0.60, AME–PME 0.15. Labium 2.45 long and 1.26 wide. Sternum 5.15 long, 1.81 wide. Palp 20.19 (7.41 + 3.65 + 4.72 +4.41), leg I 25.15 (8.71 + 4.49 + 4.59 + 4.74 + 2.62), leg II 26.62 (9.09 + 4.52 + 4.81 + 5.24 + 2.96), leg III 28.10 (9.25 + 4.28 + 5.56 + 5.89 + 3.12), leg IV 37.53 (11.06 + 4.80 + 7.70 + 9.97 + 4.00).

***Genitalia***: poreplate almost squared; posterior stalk slightly V-shaped, with a hair situated in the center dorsally; central dorsal opening small, situated in center of poreplate; receptacular cluster spherical (Fig. 3H, I).

##### Etymology.

The species epithet “hatyai” refers to the location of the type locality in Hat Yai District.

##### Distribution.

Southern Thailand (Songkhla Province) (Fig. 2).

##### Remarks.

*Liphistiushatyai* sp. nov. can be assigned to the *trang*-group according to the morphology of male palp and female genitalia. In males, the sclerotised part of embolus has two or three longitudinal ridges reaching the tip, the cumulus is slightly elevated, and the subtegulum is lacking an apophysis (Fig. 3A–G). In females, the poreplate has a small central dorsal opening and a receptacular cluster (Fig. 3H, I).

Specifically, *L.hatyai* sp. nov. belongs to the species complex D of the *trang*-group (sensu Schwendinger 1998; Schwendinger et al. 2019) based on male palp and female genital morphology. In males, the slightly elevated cumulus possesses long, needle-like spines (Fig. 3A–C), and the sclerotised part of embolus carries three longitudinal ridges reaching the tip (Fig. 3A). Female genitalia consist of a nearly squared poreplate, and a narrow, slightly V-shaped posterior stalk (Fig. 3H, I).

Species complex D includes species distributed in southern Thailand, western Peninsular Malaysia, and Sumatra. This species complex in Thailand includes *L.albipes*, *L.bicoloripes*, *L.castaneus*, *L.niphanae*, *L.trang* Platnick & Sedgwick, 1984, and *L.yangae* (for details see Schwendinger 1998; Schwendinger et al. 2019).

#### ﻿ Liphistius inthanon

Taxon classificationAnimaliaAraneaeLiphistiidae

Zhan & Xu sp. nov.

8ED5CC13-C4B0-57BD-A21F-CA0D7BBB5EFB

http://zoobank.org/59FA29B4-17FA-4613-9689-E6F639EBB8A5

Fig. 4

##### Type material.

***Holotype***: Thailand • 1 ♂, Chiang Mai Province, Mae Chaem District, Doi Inthanon National Park, 18.52°N, 98.49°E; alt. 1700 m; 19 November 2017; F.X. Liu, D. Li, X. Xu, V. Sivayyapram leg.; XUX-2017-372A. ***Paratypes***: Thailand • 1 ♂ 7 ♀♀, alt. 1700–1714 m, same data as for the holotype; XUX-2017-373A, XUX-2017-372, 374, 377, 378, 379, 380, 381.

##### Diagnosis.

The male of *L.inthanon* sp. nov. resemble males of *L.bristowei* Platnick & Sedgwick, 1984, *L.lannaianus* Schwendinger, 1990, *L.maewongensis* Sivayyapram, Smith, Weingdow & Warrit, 2017, *L.marginatus* Schwendinger, 1990 and *L.yamasakii* Ono, 1988 in having adjoining embolic parts (Fig. 4E, G) and a distinctly elevated cumulus (Fig. 4C), but it can be distinguished from the male of *L.bristowei* in having the tibial apophysis with more stouter spines (Fig. 4A–C) and a larger subtegular apophysis (Fig. 4D–G); from males of *L.lannaianus*, *L.maewongensis*, and *L.marginatus* in having the cumulus more elevated (Fig. 4C); from the male of *L.yamasakii* in having the elevated cumulus longer and with fewer spines (Fig. 4B, C); from males of *L.hatyai* sp. nov. and *L.keeratikiati* sp. nov. in having the tibial apophysis with shorter setae (Fig. 4A–C), the cumulus noticeably elevated (Fig. 4C), and a larger subtegular apophysis (Fig. 4F); from males of other *Liphistius* species in having adjoining embolic parts (Fig. 4E, G) and a strongly elevated cumulus (Fig. 4C).

The female of *L.inthanon* sp. nov. differ from the female of *L.bristowei* in having the poreplate with an arched anterior margin (Fig. 4H–M); from females of *L.lannaianus* and *L.yamasakii* in having the central dorsal opening larger oval (Fig. 4H–J); from the female of *L.maewongensis* in having the genital atrium with a wider posterior margin (Fig. 4L, M); from females of *L.hatyai* sp. nov. and *L.keeratikiati* sp. nov. in having a wider posterior stalk (Fig. 4I, J), longer oval central dorsal opening (Fig. 4H–J), and larger receptacular cluster (Fig. 4K–M); from females of other *Liphistius* species in having a wider posterior stalk (Fig. 4H–M).

##### Description.

**Male** (holotype). Carapace reddish brown, with a few short, scattered bristles; opisthosoma olive-green, with 12 dark tergites, close to each other, 2–6 larger than others, fifth largest; chelicerae robust, promargin of cheliceral groove with 9 denticles of variable size; labium yellowish brown, separated from sternum; sternum yellowish brown, with a few weakly setae on the anterior tip and many long setae on the elongated posterior tip; legs dark brown, and with strong setae and spines, without distinct annulations and with 3 tarsal claws; 8 spinnerets. Measurements: BL 17.38, CL 9.25, CW 9.62, OL 7.42, OW 5.66; eye sizes and interdistances: AME 0.18, ALE 0.87, PME 0.49, PLE 0.63, AME–AME 0.09, AME–ALE 0.22, PME–PME 0.12, PME–PLE 0.15, ALE–PLE 0.10, ALE–ALE 0.15, PLE–PLE 0.51, AME–PME 0.13. Labium 1.00 long and 0.51 wide. Sternum 4.49 long and 1.35 wide. Leg I 28.45 (7.56 + 4.16 +6.37 + 6.50 + 3.86), leg II 31.13 (8.54 + 4.01 + 6.63 + 7.70 + 4.25), leg III 34.09 (8.54 + 4.24 + 6.58 + 9.61 + 5.12), leg IV 42.23 (10.62 + 3.78 + 8.60 + 12.70 + 6.53).

***Palp***: tibial apophysis with 4 stouter spines, and several strong spines on subterminal ledge (Fig. 4A–C); paracymbium with many short strong setae situated at the tip (Fig. 4B, C); cumulus distinctly elevated with several spines on tip (Fig. 4C); subtegular apophysis large, strongly developed (Fig. 4D, F); proximal edge of contrategulum elevated (Fig. 4F, G); tegulum lunate, with dentate margin (Fig. 4G); embolic parts adjoining (Fig. 4E, F, G); embolus with 2 longitudinal ridges reaching the tip distally (Fig. 4D, E, G).

**Female** (XUX-2017-372). Carapace reddish brown, with a few short, scattered bristles; opisthosoma olive-green, with 12 dark brown tergites, close to each other, 2–6 larger than others, fifth largest; 8 eyes on dark ocular tubercle; chelicerae robust, reddish brown; promargin of chelicerae groove with 12 denticles of variable size; labium reddish brown, fused with sternum; sternum reddish brown and with several setae; legs reddish brown, with strong setae and spines, without distinct annulations, with 3 tarsal claws; 8 spinnerets. Measurements: BL 30.6, CL 12.18, CW 11.74, OL 17.49, OW 17.38; eye sizes and interdistances: AME 0.14, ALE 1.02, PME 0.52, PLE 0.82, AME–AME 0.19, AME–ALE 0.25, PME–PME 0.09, PME–PLE 0.16, ALE–PLE 0.13, ALE–ALE 0.16, PLE–PLE 0.66, AME–PME 0.12. Labium 2.78 long and 1.85 wide. Sternum 5.15 long, 1.81 wide. Palp 22.82 (7.08 + 4.24 + 5.87 + 5.63), leg I 26.61 (8.27 + 4.51 + 5.71 + 4.80 + 3.32), leg II 26.59 (7.85 + 4.96 + 5.44 + 5.16 + 3.18), leg III 28.88 (8.66 + 4.70 + 5.41 + 6.41 + 3.70), leg IV 33.46 (9.37 + 3.76 + 7.23 + 8.79 + 4.31).

***Genitalia***: poreplate with a long, oval central dorsal opening, and with projecting posterior corners; receptacular cluster racemose and large; posterior stalk wide, lateral margins of genital atrium with some hairs (Fig. 4H–M).

##### Etymology.

The species epithet “inthanon” is a toponym referring to the type locality, Doi Inthanon National Park.

##### Distribution.

Northern Thailand (Chiang Mai Province) (Fig. 2).

##### Variation.

The range of females’ measurements (*N* = 7): BL 16.92–30.6, CL 7.90–12.18, CW 7.53–11.74, OL 8.5–17.49, OW 6.63–17.38. The number of denticles on the promargin of cheliceral groove varies from 12–14 (*N* = 7). The examined female genitalia were found to differ in that the posterior margin of genital atrium can be narrow, slightly W-shaped (Fig. 4H, K), or wide and straight (Fig. 4I, J, L, M), and the shape of poreplate anterior margin can slightly vary (Fig. 4K–M).

##### Remarks.

*Liphistiusinthanon* sp. nov. can be assigned to the *bristowei*-group based on the following characters: the male palp has a pronounced, elevated cumulus (Fig. 4C); the embolic parts are adjoining (Fig. 4D, G), the sclerotised part of the embolus bears two longitudinal ridges reaching the tip (Fig. 4D, G), and, except for *L.marginatus*, all have a large subtegular apophysis (Fig. 4F); the poreplate has a wide posterior stalk and a projecting posterior corner (Fig. 4H–M). The *bristowei*-group contains *L.bristowei*, *L.lannaianus*, *L.maewongensis*, *L.marginatus*, *L.yamasakii* (Schwendinger 1990), and *L.inthanon* sp. nov.

#### ﻿ Liphistius keeratikiati

Taxon classificationAnimaliaAraneaeLiphistiidae

Zhan & Xu sp. nov.

7CC66D8D-4334-550A-A29F-171C9CE226DD

http://zoobank.org/A45A1921-8728-4095-9496-EBBED27DD903

Fig. 5

##### Type material.

***Holotype***: Thailand • 1 ♂, Chumphon Province, Sawi District, Khao Thalu Subdistrict, Nam Lot Cave. 10.23°N, 98.94°E; alt. 30 m; 25 November 2017; F.X. Liu, D. Li, X. Xu, V. Sivayyapram leg.; XUX-2017-439. ***Paratypes***: Thailand • 1 ♂, 3♀♀, same data as for the holotype; XUX-2017-439, XUX-2017-431, 436, 438.

##### Diagnosis.

The male of *L.keeratikiati* sp. nov. can be distinguished from the male of *L.fuscus* Schwendinger, 1995 in having the paraembolic plate scale-like and arched (Fig. 5A, E), and the tibial apophysis slightly wider basally (Fig. 5A–C), while in *L.fuscus* the paraembolic plate is broadly rounded; from the male of *L.phuketensis* Schwendinger, 1998 in having the tibial apophysis with four setae (Fig. 5B, C); from the male of *L.schwendingeri* Ono, 1988 in having a longer embolus (Fig. 5A, B, E), the contrategulum with fewer wrinkles proximally (Fig. 5G), and a smaller tegulum (Fig. 5B, C, G); from the male of *L.hatyai* sp. nov. in having the tibial apophysis with longer setae and the paracymbium narrower (Fig. 5A–C); from the male of *L.inthanon* sp. nov. in having the subtegular apophysis absent (Fig. 5B, F) and the paraembolic plate scale-like (Fig. 5E); from males of other *Liphistius* species in having the spines on the cumulus slightly separated from setae on the paracymbium (Fig. 5A–C).

The female of *L.keeratikiati* sp. nov. differs from the female of *L.fuscus* in having the anterior margin of the poreplate slightly curved (Fig. 5H, I); from the female of *L.phuketensis* in having the anterior margin of the poreplate slightly narrower (Fig. 5I); from the female of *L.schwendingeri* in having the receptacular cluster slightly larger (Fig. 5I); from females of other *Liphistius* species in having the arched poreplate lacking lateral edges (Fig. 5I, J) and much wider than long (Fig. 5H, I), and the central dorsal opening situated in the lower center of the poreplate (Fig. 5H).

##### Description.

**Male** (holotype). Carapace light yellow, with a few short, scattered bristles; opisthosoma yellow, with 12 tergites, with light brown patches; close to each other, 2–6 larger than others, fifth largest; chelicerae robust, promargin of cheliceral groove with 6 denticles of variable size; labium yellow and separated from sternum; sternum yellow, with a few short setae on anterior tip and many long setae on the elongated posterior tip; legs with strong setae and spines; with white annulations, with 3 tarsal claws; 8 spinnerets. Measurements: BL 15.61, CL 6.92, CW 6.72, OL 7.24, OW 5.07; eye sizes and interdistances: AME 0.09, ALE 0.74, PME 0.43, PLE 0.54, AME–AME 0.12, AME–ALE 0.15, PME–PME 0.03, PME–PLE 0.10, ALE–PLE 0.08, ALE–ALE 0.05, PLE–PLE 0.38, AME–PME 0.07. Labium 1.04 long and 0.76 wide. Sternum 3.02 long, 0.95 wide. Leg I 21.60 (5.41 + 1.81 + 4.68 + 4.79 + 4.91), leg II 23.29 (4.97 + 2.19 + 7.68 + 5.96 + 2.49), leg III 14.64 (missing metatarsus and tarsus) (6.17 +2.87 + 5.60 + NA + NA), leg IV 30.16 (7.69 + 3.08 + 5.97 + 9.31 + 4.11).

***Palp***: tibial apophysis pronounced elevated, with four tapering spines of similar length (Fig. 5A–C); paracymbium with short, strong setae situated at tip (Fig. 5C), and 5 tapering spines on elevated cumulus (Fig. 5A, B); subtegulum without apophysis (Fig. 5B, F); contrategulum with a process distally, and with several wrinkles proximally (Fig. 5B, G); tegulum with a dentate edge (Fig. 5B, G); embolic parts detached (Fig. 5B), paraembolic plate scale-like, semicircular (Fig. 5A, E); embolus slender, with a few denticulations at the tip (Fig. 5A, B, G).

**Female** (XUX-2017-431). Carapace light brown, with few short, scattered bristles; opisthosoma gray, with 12 brown tergites, close to each other, 2–6 larger than others, fifth largest; eight eyes on darkened ocular tubercle; chelicerae robust, brown, promargin of chelicerae groove with 11 denticles of variable size; labium yellow, separated from sternum; sternum yellow with several setae; legs with strong hairs and spines; with brown and yellow annulations and 3 tarsal claws; 8 spinnerets. Measurements: BL 16.9, CL 7.21, CW 6.67, OL 9.93, OW 7.57; eye sizes and interdistances: AME 0.07, ALE 0.64, PME 0.30, PLE 0.51, AME–AME 0.08, AME–ALE 0.16, PME–PME 0.06, PME–PLE 0.09, ALE–PLE 0.09, ALE–ALE 0.08, PLE–PLE 0.41, AME–PME 0.09. Labium 1.49 long and 0.76 wide. Sternum 3.40 long, 1.13 wide. Palp 13.37 (4.74 + 2.25 + 3.32 + 3.06), leg I 16.58 (5.65 + 2.72 + 3.62 + 2.92 + 1.67), leg II 19.13 (5.56 + 3.06 + 3.76 + 3.71 + 2.05), leg III 17.14 (4.99 + 2.83 + 3.91 + 3.92 + 2.29), leg IV 24.73 (6.82 + 2.31 + 5.39 + 7.03 + 3.18).

***Genitalia***: poreplate much wider than long, arched (Fig. 5J), lateral edges absent (Fig. 5I); central dorsal opening situated in the lower center of poreplate (Fig. 5H); receptacular cluster simple (Fig. 5I).

##### Etymology.

The specific name is dedicated to Mr Kaweesak Keeratikiat for providing information on the locality of the species.

##### Distribution.

South-central Thailand (Chumphon Province) (Fig. 2).

##### Variation.

Range in female measurements (*N* = 3): BL 16.45–18.89, CL 7.15–7.49, CW 6.45–7.15, OL 8.79–10.46, OW 6.76–8.99. The number of denticles on the promargin of cheliceral groove varies from 6–13 (*N* = 3).

##### Remarks.

*Liphistiuskeeratikiati* sp. nov. can be assigned to the *trang*-group according to the morphology of male palp and female genitalia, see the remarks of *hatyai* sp. nov. The new species can be assigned to the species complex C of the *trang*-group. In males, the palp possesses the contrategulum with wrinkles proximally (Fig. 5B, G), the tegulum has a dentate edge (Fig. 5C, F, G), the spines on the elevated cumulus are slightly, distinctly separated from the setae on the paracymbium, and the apex of the embolus bears a few denticulations (Fig. 5A–C, E, G). In females, the poreplate is lacking lateral edges (Fig. 5I), arched (Fig. 5J), wider than long (Fig. 5H–I), and lacking a posterior stalk (Fig. 5H, I). Currently, the species complex C contains *L.fuscus*, *L.phuketensis*, *L.schwendingeri* (Schwendinger et al. 2019), and *L.keeratikiati* sp. nov.

## Supplementary Material

XML Treatment for Liphistius

XML Treatment for Liphistius hatyai

XML Treatment for Liphistius inthanon

XML Treatment for Liphistius keeratikiati
